# Glial Fibrillary Acid Protein Reflects Disease Activity in Autoimmune Encephalitis

**DOI:** 10.1111/ene.70207

**Published:** 2025-05-23

**Authors:** Johannes Piepgras, Marlene L. Piepgras, Falk Steffen, Laura Ehrhardt, Martin A. Schaller‐Paule, Yavor Yalachkov, Frauke Zipp, Stefan Bittner

**Affiliations:** ^1^ Department of Neurology, Focus Program Translational Neuroscience (FTN) and Immunotherapy (FZI), Rhine‐Main Neuroscience Network (rmn2) University Medical Center of the Johannes Gutenberg University Mainz Mainz Germany; ^2^ Mainz Germany; ^3^ Department of Pediatrics University Medical Center of the Johannes Gutenberg University Mainz Mainz Germany; ^4^ Department of Neurology Goethe University, University Hospital Frankfurt Frankfurt am Main Germany

**Keywords:** autoimmune encephalitis, biomarker, glial fibrillary acid protein, neurofilament, neuroinflammation

## Abstract

**Background and Purpose:**

Management of autoimmune encephalitis (AE) is challenging due to a lack of reliable biomarkers. We here assess the combination of glial fibrillary acid protein (GFAP) and neurofilament (NfL) as biomarkers for diagnosis and disease monitoring of AE.

**Methods:**

GFAP and NfL CSF levels (cGFAP, cNfL) of 42 AE patients were correlated with CSF markers of neuroinflammation. NfL/GFAP ratios were compared between patients with stable and active AE, stable and active multiple sclerosis (MS), and patients undergoing diagnostic lumbar puncture without evident pathological alterations (controls).

**Results:**

In patients with AE, cGFAP levels showed strong correlations with albumin and IgG quotients and moderate correlations with CSF cell count; cNfL levels showed weak correlations with albumin quotients. cGFAP and cNfL levels showed no significant differences between patients with and without epileptic activity or inflammatory MRI lesions. Both sNfL and sGFAP correlated with the Clinical Assessment Scale in Autoimmune Encephalitis. Compared to NfL or GFAP alone, the NfL/GFAP ratio from CSF or serum led to a clearer separation of AE from MS patients and controls. Furthermore, serum NfL/GFAP ratios better discriminated active from stable AE.

**Conclusion:**

cGFAP levels indicate intrathecal inflammatory processes in patients with active AE to a stronger degree than cNfL levels. Serum NfL/GFAP ratios recognize active AE, suggesting this ratio identifies AE patients with CNS‐compartmentalized neuronal injury (autoantibody‐mediated or cytotoxic) behind a relatively intact blood–brain barrier. Our findings indicate that the NfL/GFAP ratio can function as a blood‐based biomarker, aiding clinicians with diagnosis and disease management of AE.

## 1 Introduction

Autoimmune encephalitis (AE) is an immune‐mediated condition that is highly heterogeneous in clinical presentation and provides challenges to diagnosis and monitoring due to a lack of reliable biomarkers. Symptoms can vary, creating a spectrum of syndromes including epileptic seizures, motor and coordination deficits, as well as cognitive and psychiatric deficits such as dementia‐like and psychotic episodes affecting children and adults. Either by a predominant autoantibody‐mediated or cytotoxic neuroimmunological phenotype, AE leads to neuronal dysfunction, which can rapidly progress to irreversible neurodegeneration leading to persisting neurological and/or psychiatric deficits if untreated. Currently, patients with suspected AE undergo cranial magnetic resonance imaging (MRI) and lumbar puncture, which—especially for children and psychotic patients—often is inaccessible. Pitfalls in diagnostic work‐up often include inconclusive or negative autoantibody detection due to test methodology [1] and cranial MRI scans can present normal even in acute disease [2, 3]. Neuroinflammatory cerebrospinal fluid (CSF) changes, such as CSF pleocytosis, intrathecal IgG synthesis, and blood–brain barrier breakdown are a common occurrences in AE and independent of the underlying pathomechanism.

Neurofilament (NfL) and glial fibrillary acid protein (GFAP), cytoskeletal proteins of central nervous system (CNS) neurons and astrocytes, respectively, can be detected by blood‐based tests. NfL has been studied in various CNS diseases, especially multiple sclerosis (MS), where it is gaining increasing importance [4]. While some studies in AE describe an association of NfL with disease severity, measured as correlation with the Modified Rankin Scale (mRS) or inflammatory MRI lesions, and attribute NfL a predictive value, other publications do not confirm this association, much less a predictive potential [5, 6, 7, 8, 9, 10, 11].

In MS, GFAP is currently discussed as an additional biomarker reflecting disease progression independent of inflammatory activity [12, 13, 14]. Regarding AE, to date, there is only one older study in which GFAP was analyzed in CSF (cGFAP) in a small and heterogeneous AE patient cohort, and no association with disease activity was found [7]. Studies on serum GFAP (sGFAP) with current single molecule arrays (SiMoA) and in larger AE patient cohorts are still lacking.

Thus, the aim of our study was to evaluate differences in GFAP and NfL levels in AE and analyze their combination as easily accessible biomarkers in an AE patient cohort. To evaluate the potential of GFAP and NfL to resolve different pathophysiological patterns of neuroinflammation, we correlated CSF GFAP and NfL levels with CSF markers of neuroinflammation. CSF and serum GFAP and NfL levels and corresponding NfL/GFAP ratios were compared between patients with active AE, active progressive MS, stable MS, and patients undergoing diagnostic lumbar puncture without evident pathological alterations (controls). Furthermore, we applied sNfL/sGFAP ratios to differentiate active from inactive AE patients. Our findings indicate that the ratio of NfL/GFAP can serve clinicians as a blood‐based biomarker for diagnosis and disease management of AE.

## 2 Methods

### 2.1 Participants

Patients were retrospectively recruited at the Department of Neurology of the University Medical Center Mainz (Mainz, Germany) and University Hospital Frankfurt (Frankfurt, Germany) from 2010 to 2023. The study was approved by the local ethics committee. All patients gave written informed consent in accordance with ethical standards of the 1964 Helsinki Declaration and its later amendments and comparable ethical standards. Patient characteristics are summarized in Table 1. Modified Rankin Scale (mRS) and Clinical Assessment Scale in Autoimmune Encephalitis (CASE) were estimated retrospectively using medical reports.

**TABLE 1:** Characteristics of AE and MS patients.

	Autoimmune encephalitis	Active multiple sclerosis	Multiple sclerosis NEDA3	Controls
Number of patients, *n*	42	31	21	20
PMS/RRMS, *n*/*n*	n.a.	31/0	9/12	n.a.
Number of CSF samples, *n*	48	3	7	19
Number of serum samples, *n*	81	28	14	20
Age, years, median (SD)	56 (19)	51 (10)	50 (12)	33 (10)
Female/male, *n*/*n*	23/19	19/12	14/7	15/5
Active/inactive, *n*/*n*	31/11	31/0	0/21	n.a.

Abbreviations: CSF, cerebrospinal fluid; *n*, number; n.a., not applicable; NEDA3, no evidence of disease activity; PMS, progressive multiple sclerosis; RRMS, relapsing–remitting multiple sclerosis; SD, standard deviation.

### 2.2 Single Molecule Array Analysis of NfL and GFAP Levels

Blood and CSF samples were centrifuged at 2500 × g for 10 min at 4°C. Supernatant was stored at −80°C. CSF and serum NfL and GFAP levels were determined using the highly sensitive SiMoA technology. Samples were measured in duplicates with the SiMoA HD‐1 (Quanterix, USA) using NF‐Light Advantage and GFAP Discovery kits according to the manufacturer's instructions. Mean inter‐assay and intra‐assay coefficients of variation were less than 10%. Measurements were performed blinded without information about clinical data.

### 2.3 Statistical Analysis

To determine whether GFAP and NfL values from CSF and serum correlate with one another, or whether cGFAP and cNfL correlate with CSF markers for neuroinflammation, we performed age‐corrected partial correlations. Mann–Whitney *U* Tests were performed to analyze whether cNfL and cGFAP values differ between AE patients in the presence or absence of cranial inflammatory MRI lesions or epileptic seizures, and active or inactive disease. Kruskal‐Wallis Tests were performed to compare CSF and serum levels of NfL, GFAP, and NfL‐GFAP ratio between active, stable AE, active chronic MS, stable MS patients, and controls. Values are described as median with interquartile range (IQR, 25th and 75th percentiles). Statistical significance was assumed if *p*‐values were equal to or less than 0.05 (*), 0.005 (**) or 0.001 (***), respectively. Statistical analysis was performed using IBM SPSS Statistics software Version 27.0.1.0.

## 3 Results

### 3.1 Clinical Characteristics of AE and MS Patients

In total, 114 patients participated in this retrospective study, including patients suffering from AE (*n* = 42), MS (*n* = 52), and controls (*n* = 20). There was no significant difference in age between the AE and MS patient cohorts. AE patients fulfilled criteria proposed by Graus et al. [15]. Targets of neuronal autoantibodies included NMDAR, LGI1, CASPR2, DPPX, AMPA, GABAa, IgLON5, GAD, MOG, Hu, and Ma/Ta2. AE patients were considered to have active disease (*n* = 31/42) if one or more of the following criteria were met: new neurological or psychiatric symptoms, gadolinium‐enhancing MRI brain lesions, new non‐gadolinium‐enhancing MRI brain lesions, elevated CSF cell count, elevated IgG ratio (QIgG) or elevated albumin ratio (QAlb, an indicator of blood–brain barrier integrity); otherwise, they were considered to have stable disease (*n* = 11/42). Similarly, MS patients were allocated to the active disease group (*n* = 31/52) if one or more of the following criteria were met: EDSS progression, new inflammatory MRI alterations, or new clinical relapses. MS patients were regarded as stable (*n* = 21/52) if none of the criteria were met (termed no evidence of disease activity, NEDA3). Patient characteristics are summarized in Tables 1 and 2.

**TABLE 2:** AE patient cohort including demographics, antibody subtype, presence of tumors, MRI activity, epileptic activity, and maximum (worst) CASE and mRS scores.

	Surface abs	Intracellular abs	Ab negative
Number of patients, *n*	18	7	17
Female/male, *n*/*n*	12/6	3/4	8/9
Age, years, median (SD)	46 (23)	61 (14)	61 (13)
Tumor present, *n*	0	2	3
Inflammatory MRI present, *n*	8	7	9
Epileptic activity present, *n*	9	3	7
CASE, score, media (SD)	5 (3)	4 (2)	5 (2)
mRS, score, median (SD)	3 (1)	3 (1)	3 (1)

Abbreviations: ab, antibody; SD, standard deviation.

### 3.2 CSF GFAP Levels Indicate Intrathecal Inflammatory Processes in AE

**FIGURE 1:**
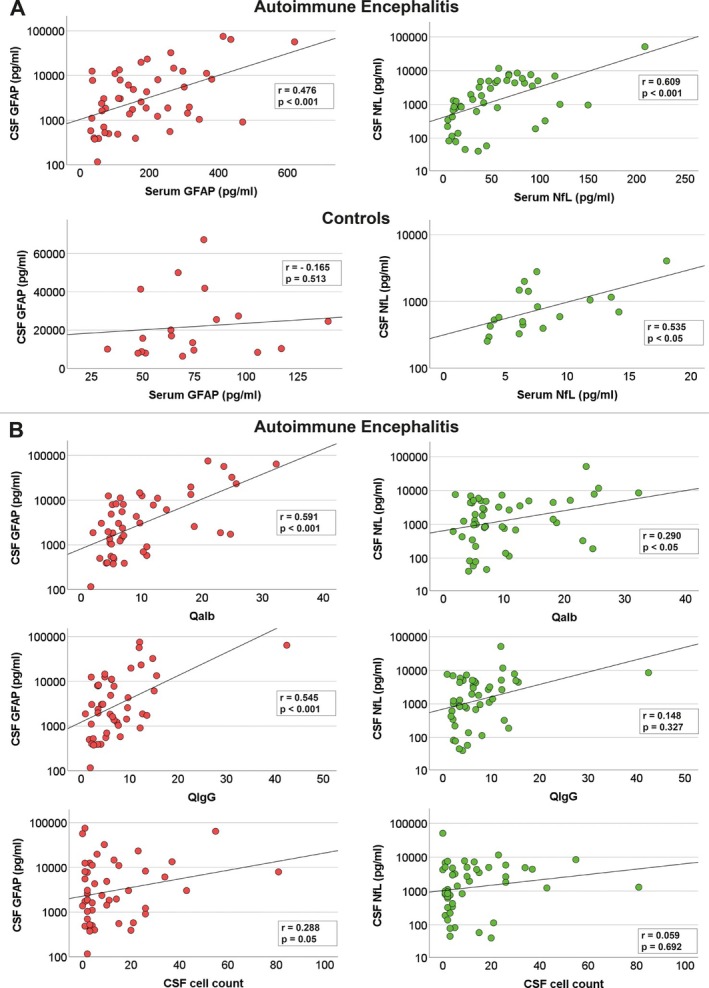
CSF GFAP indicates brain inflammation in AE. (A) Age‐corrected partial correlation analysis was performed with paired CSF and serum values of 46 AE patients and 20 controls. Whereas CSF and serum GFAP and NfL levels showed a robust correlation in AE patients, in the control group, only CSF and serum NfL levels correlated. (B) Age‐corrected partial correlation analysis of cGFAP and cNfL values with corresponding values of albumin and IgG quotients, and CSF cell count was performed, showing a robust correlation of cGFAP with Qalb and QIgG. cNfL showed moderate correlation with Qalb and no correlation with intrathecal IgG synthesis. Correlation with CSF cell count showed moderate correlation for cGFAP and no correlation for cNfL.

To determine whether levels of both markers in blood samples represent ongoing disease activity in the brain, we used age‐corrected partial correlation analysis of paired CSF and serum samples from the AE cohort, which revealed robust correlation of cGFAP with sGFAP and cNfL with sNfL (GFAP: *r* = 0.476, *p* < 0.001; NfL: *r* = 0.609, *p* < 0.001). In the control group, NfL, but not GFAP, showed correlation of CSF with serum values (GFAP: *r* = − 0.165, *p* = 0.513; NfL *r* = 0.535, *p* < 0.05; Figure 1A). Evaluating whether both markers represent ongoing brain inflammation, age‐corrected partial correlation analysis of cGFAP and cNfL with QAlb, QIgG, and cell count revealed strong correlations of cGFAP with QAlb as a marker of blood–brain barrier integrity (*r* = 0.591, *p* < 0.001) and intrathecal IgG synthesis (*r* = 0.545, *p* < 0.001), while cNfL showed moderate correlation with QAlb (*r* = 0.290, *p* < 0.05), and no correlation with intrathecal IgG synthesis (*r* = 0.148, *p* = 0.327). Correlation with CSF cell count showed moderate correlation for cGFAP (*r* = 0.288, *p* = 0.05), and no correlation for cNfL (*r* = 0.059, *p* = 0.692; Figure 1B). These findings indicate that while CSF and serum levels of both NFL and GFAP correlate in patients with AE, intrathecal inflammation influences GFAP levels to a stronger extent than NFL in patients with AE.

### 3.3 CSF GFAP and NfL Are Not Affected by Epileptic Activity or Inflammatory MRI Lesions in AE

Group comparison analysis revealed that cGFAP and cNfL remained unaffected by the presence or absence of epileptic activity (cGFAP: median 2473.58 pg/mL, IQR 1137.03–11171.40 in patients without epileptic activity vs. 2506.2 pg/mL, IQR 943.8–7912.02 with epileptic activity, *p* = 0.836; cNfL: median 1887.52 pg/mL, IQR 302.06–4799.02 in patients without epileptic activity: vs. 1270.2 pg/mL, IQR 815.18–4685.36 with epileptic activity, *p* = 0.836). Since inflammatory lesions in cranial MRI scans may be absent in AE patients and hence AE diagnosis can be missed by an MRI‐based diagnostic workup, we also performed group comparison analysis between patients with and without inflammatory MRI lesions. The results showed no significant difference between both groups (cGFAP: median 1879.38 pg/mL, IQR 567.49–9419.5 in patients without inflammatory MRI lesions vs. 3043.27 pg/mL, IQR 1224.02–8269.85 with inflammatory MRI lesions, *p* = 0.480; cNfL: median 680.92 pg/mL, IQR 164.61–4260.19 in patients without inflammatory MRI lesions vs. 1960.61 pg/mL, IQR 875.05–4790.13 with inflammatory MRI lesions, *p* = 0.107). Our results indicate that cGFAP and cNfL levels are stable markers that are not confounded by concomitant epileptic activity or MRI lesion burden in AE.

### 3.4 CSF and Serum NfL/GFAP Ratio Differentiate AE From Other Inflammatory CNS Diseases

**FIGURE 2:**
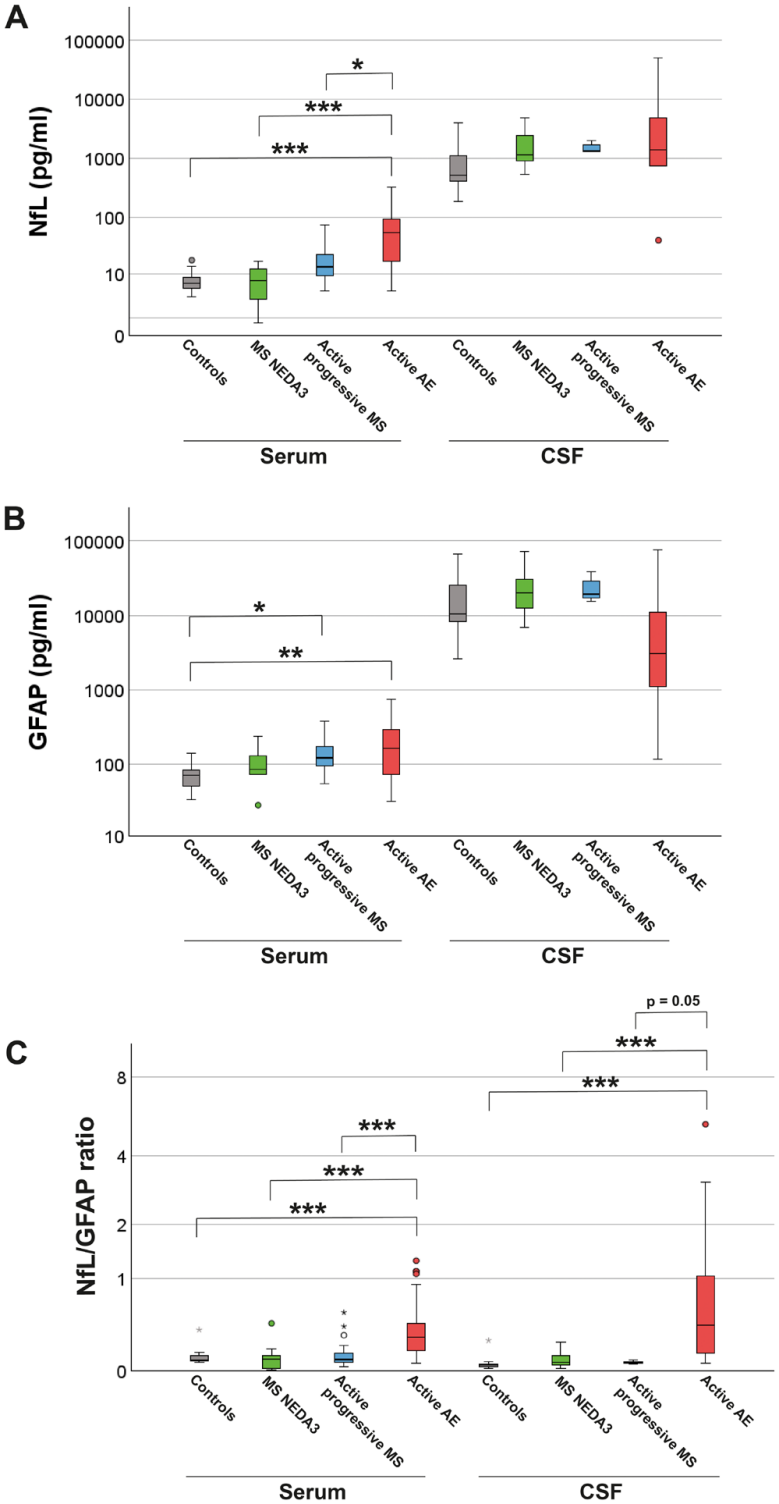
The serum NfL/GFAP ratio discriminates AE from other inflammatory CNS diseases and controls. Differences in NfL (A), GFAP (B), and corresponding NfL/GFAP ratio levels (C) of controls, MS patients with no evidence of disease activity (NEDA3), active progressive MS patients, and active AE patients were analyzed by Kruskal‐Wallis test. Calculation of the sNfL/sGFAP ratio led to clearer separation of AE from MS patients and controls compared to the use of mere sNfL or sGFAP values. Additionally, calculation of the cNfL/cGFAP ratio was able to separate AE patients from controls and MS NEDA3 patients; *p < 0.05, **p < 0.01, ***p < 0.001.

To address the hypothesis that GFAP and NfL show different patterns across inflammatory CNS diseases, we compared their CSF and serum levels between controls, stable (NEDA3) MS patients, active progressive MS patients, and active AE patients. sNfL values were highest in active AE patients (median 55.22 pg/mL, IQR 17.78–93.07) and lowest in controls (median 6.7 pg/mL, IQR 5.71–8.38) and stable MS patients (median 7.62 pg/mL, IQR 3.27–12.7). Active progressive MS patients showed intermediate sNfL levels (median 13.55 pg/mL, IQR 9.72–22.22; active AE vs. active progressive MS *p* < 0.5, active AE vs. MS NEDA3 *p* < 0.001, active AE vs. controls *p* < 0.001, active progressive MS vs. MS NEDA3 *p* = 0.149, active progressive MS vs. controls *p* = 0.069 and MS NEDA3 vs. controls *p* = 0.865; Figure 2A). sGFAP levels were highest in AE patients (median 165.45 pg/mL, IQR 75.39–290.91) and comparable between controls (median 71.7 pg/mL, IQR 50.96–81.4), stable (median 85.24 pg/mL, IQR 72.6–130.38), and active progressive MS patients (median 122.83 pg/mL, IQR 94.40–171.7; active AE vs. active progressive MS *p* = 0.694, active AE vs. MS NEDA3 *p* = 0.108, active AE vs. controls *p* < 0.01, active progressive MS vs. MS NEDA3 *p* = 0.246, active progressive MS vs. controls *p* < 0.05 and MS NEDA3 vs. controls *p* = 0.345; Figure 2B). sNfL/sGFAP ratios (generated from corresponding sNfL and sGFAP values) were highest in active AE patients (median 0.28, IQR 0.16–0.42) and significantly lower in controls (median 0.08, IQR 0.08–0.12), stable MS patients (median 0.09, IQR 0.02–0.11), and active progressive MS patients (median 0.09, IQR 0.07–0.14; active AE vs. MS NEDA3 *p* < 0.001, active AE vs. active progressive MS *p* < 0.001, active AE vs. MS NEDA3 *p* < 0.001, active AE vs. controls *p* < 0.001, active progressive MS vs. MS NEDA3 *p* = 0.424, active progressive MS vs. controls *p* = 0.828, and MS NEDA3 vs. controls *p* = 0.57; Figure 2C). Interestingly, the sNfL/sGFAP ratio led to a clearer separation of active AE patients from controls and MS patients. We also compared cGFAP, cNfL, and cNfL/cGFAP ratios between controls, stable MS, active progressive MS, and active AE patients. While cNfL (median of controls 515.31 pg/mL, IQR 404.19–1132.68; median of MS NEDA3 1168.65 pg/mL, IQR 901.07–2424.91; median of active progressive MS 1334.03 pg/mL, IQR 1314.27–1681.84; median of active AE 1410.70, IQR 757.44–4921.39; *p* = 0.484) and cGFAP (median of controls 10062.25, IQR 7981.06–24581.70; median of MS NEDA3 20056.86 pg/mL, IQR 12573.71–30082.41; median of active progressive MS 19302.28 pg/mL, IQR 17358.62–28659.20; median of active AE 3082.10, IQR 1380.97–12526.44; *p* = 0.155) showed no significant differences, the cNfL/cGFAP ratio successfully separated AE from stable MS patients and controls but barely missed statistical significance in separating AE from active progressive MS patients (median of controls 0.047, IQR 0.034–0.052; median of MS NEDA3 0.06, IQR 0.04–0.12; median of active progressive MS 0.06, IQR 0.06–0.07; median of active AE 0.406, IQR 0.145–1.037; active AE vs. controls *p* < 0.001; active AE vs. MS NEDA3 *p* < 0.001; active AE vs. active progressive MS *p* = 0.05). Taken together, our findings indicate that in AE, the NfL/GFAP ratio demonstrates higher specificity than GFAP or NfL alone.

### 3.5 Serum GFAP and NfL Levels as Markers for Disease Severity in AE

**FIGURE 3:**
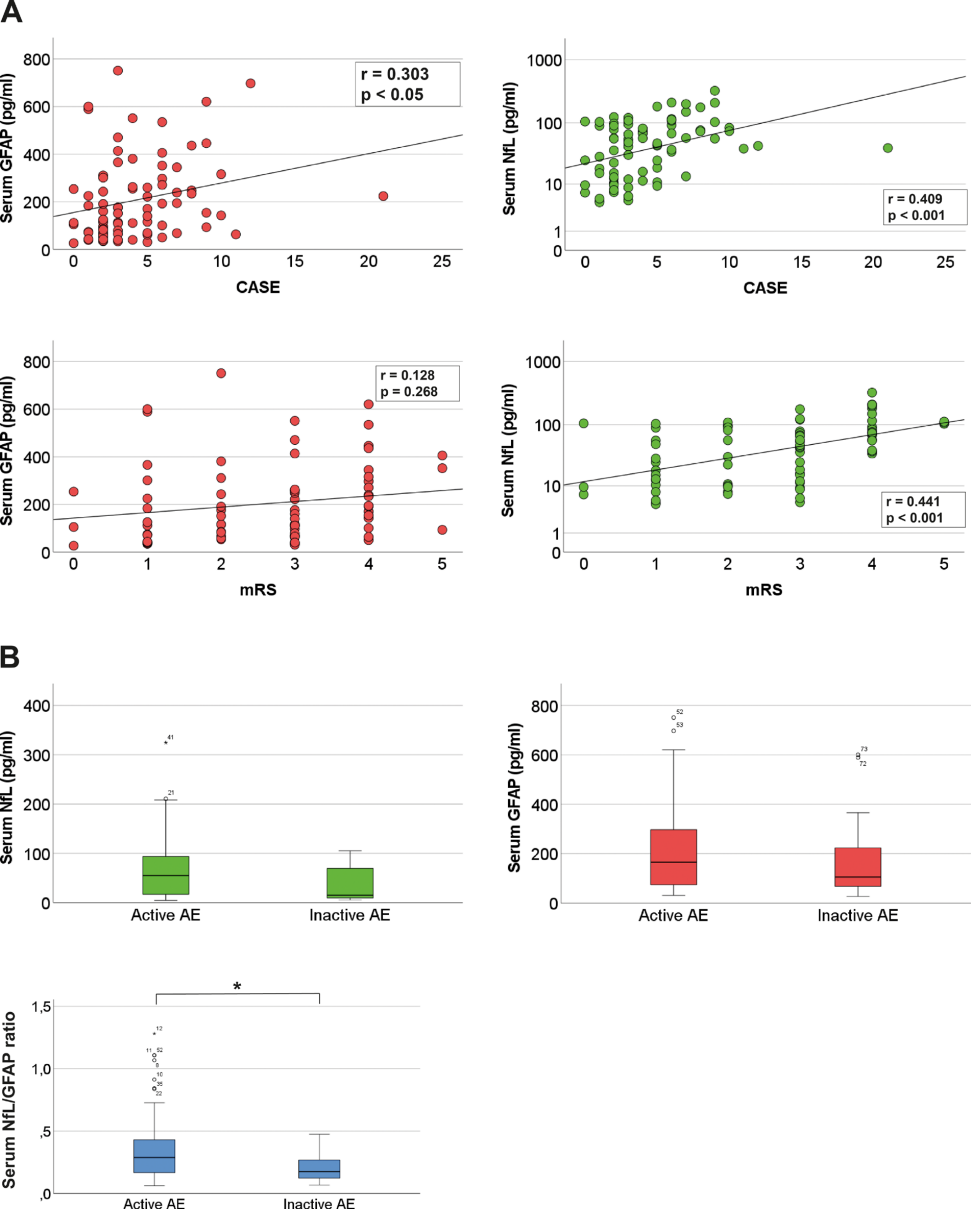
While both sNFL and sGFAP correlate with disease severity scores, a combined serum NfL/GFAP ratio best discriminates active from inactive AE. (A) Age‐corrected partial correlation analysis of sGFAP and sNfL values with corresponding values of mRS and CASE was performed showing a correlation of sGFAP with CASE, a clinical score with a greater resolution of neuronal dysfunction associated with AE. sNfL showed a robust correlation with both mRS and CASE. (B) Serum samples of AE patients with active vs. inactive disease were analyzed by the Kruskal‐Wallis test regarding their sNfL, sGFAP, and corresponding sNfL/sGFAP ratios showing a clearer separation of both patient groups by sNfL/sGFAP ratios; *p* < 0.05.

Age‐corrected partial correlation analysis was performed to analyze whether sGFAP and sNfL reflect disease severity measured by mRS and CASE. Interestingly, sGFAP values showed no correlation with mRS (*r* = 0.128, *p* = 0.268) but did correlate with CASE (*r* = 0.303, *p* < 0.05), a score tailored to the diverse spectrum of neuronal dysfunction in AE. sNfL correlated with both mRS (*r* = 0.441, *p* < 0.001) and CASE (*r* = 0.409, *p* < 0.001, Figure 3A). Furthermore, we compared sNfL/sGFAP ratios between AE patients with active versus inactive disease. While sNfL and sGFAP levels between active and inactive AE patients showed high overlap (sNfL: median 55.22 pg/mL, IQR 17.78–93.07 in active patients vs. 15.46 pg/mL, IQR 9.64–69.46 in inactive patients, *p* = 0.072; sGFAP: median 165.54 pg/mL, IQR 75.4–290.82 in active patients vs. 105.33 pg/mL, IQR 67.81–223.35 in inactive patients, *p* = 0.368), the sNfL/sGFAP ratio led to a clear separation of active from inactive AE patients (median 0.29, IQR 0.16–0.43 in active patients vs. 0.17, IQR 0.12–0.26 in inactive patients, *p* < 0.05; Figure 3B). This effect was not observed when comparing corresponding CSF levels (cNfL: median 1410.7 pg/mL, IQR 757.45–4921.4 in active AE patients vs. 429.3 pg/mL, IQR 233.3–3108.83 in inactive AE patients; cGFAP: median 3035.16 pg/mL, IQR 1108.03–11054.25 in active AE patients vs. 1876.59, IQR 600.63–3102.83 in inactive AE patients; cNfL/cGFAP ratio: median 0.41, IQR 0.15–1.04 in active AE patients vs. 0.27, IQR 0.2–0.8 in inactive AE patients). These findings suggest that both biomarkers reflect disease severity in AE and, if combined with serum NfL/GFAP ratios, will aid clinicians in differentiating between stable and active disease.

## 4 Discussion

AE presents with heterogeneous pathomechanisms, symptoms, and disease courses. We analyzed the potential of cGFAP and cNfL to reflect intrathecal inflammatory processes by correlating each biomarker with the albumin quotient, IgG quotient, and CSF cell count. Interestingly, even though underlying autoantibody pathologies differ in our 42 AE patient cohort, our findings suggest that especially cGFAP mirrors inflammatory processes within the CNS compartment. Constantinescu et al., who studied CSF markers including GFAP and NfL in a longitudinal study, did not report a correlation, which is most likely due to the group's methodological approach by not correlating cNfL and cGFAP with the albumin and IgG quotient separately [7]. Concerning NfL, the findings of Macher et al. and Nissen et al. are consistent with our results observing no correlation between cNfL and IgG quotient or CSF cell count in a small (*n* = 6) anti‐N‐methyl‐D‐aspartate receptor (NMDAR) encephalitis patient cohort [8] and an AE patient cohort consisting of anti‐NMDAR encephalitis (*n* = 37) and anti‐leucine‐rich glioma inactivated 1 (LGI1) encephalitis (*n* = 16) patients [16]. In contrast to our findings, Nissen et al. did not find an association between cNfL and blood–brain barrier integrity. This might be explained by the group's approach analyzing total CSF protein, whereas we analyzed albumin quotient as a marker for blood–brain barrier integrity. As astrocytes are the main component of the blood–brain barrier, the strong correlation of GFAP with the albumin quotient most likely relies on astrocytic activation and possibly damage during blood–brain barrier breakdown with a consecutive rise of cytoskeletal GFAP. This is supported by our observation that GFAP only showed CSF‐serum correlation in the AE patient group and not in the control group where blood–brain barrier function is unimpaired. Blood‐brain barrier dysfunction and intrathecal IgG synthesis, often happening in parallel during CNS inflammatory conditions, explain the equally strong correlation of GFAP with the IgG quotient [17, 18].

A biomarker for AE would need to be reliably interpretable even under such adverse conditions as an epileptic seizure. Moreover, cranial MRI is an important diagnostic tool in diagnosing AE, but can also be negative in acute AE. Therefore, we compared cNfL and cGFAP levels between AE patients with and without epilepsy and between MRI positive and negative AE patients. In our AE patient cohort, cNfL and cGFAP levels did not show any association with epileptic activity or MRI lesion burden. For epilepsy, available publications report high NfL levels if blood samples were drawn during status epilepticus or in patients with therapy refractory epilepsy suggesting neurodegeneration in both scenarios. On the other hand, in limited epileptic seizures, NfL and GFAP only showed slight increase compared to non‐epileptic samples [19, 20, 21, 22]. In line with this, our findings indicate that limited epileptic seizures are unlikely to confound NfL and GFAP levels when used as a diagnostic tool for AE. For MRI, our findings are in line with previous publications [7, 9, 10]. Patients with acute anti‐NMDAR encephalitis might be an exception, since Brenner et al. and Nissen et al. report elevated cNFL and sNfL values with concomitant MRI lesions [6, 8]. This heterogeneity might result from a lack of specificity regarding MRI lesions in the available studies. Thus, future studies should differentiate MRI lesions, e.g., gadolinium‐enhancing versus non‐enhancing lesions and lesion volume.

To evaluate whether combining GFAP and NfL could increase their specificity in diagnosing AE, we compared CSF and serum NfL/GFAP ratios between active AE patients and age‐matched active progressive MS patients, age‐matched inactive MS patients, and controls. Group comparison analysis revealed that sNfL/sGFAP ratios led to clearer discrimination of AE from stable and active progressive MS patients and controls. Interestingly, only cNfL/cGFAP ratios but not cGFAP or cNfL values alone were significantly different between AE patients, stable MS patients and controls. The lack of statistical significance when comparing AE with active progressive MS patients (p = 0.05) most likely results from the small number of CSF samples from active progressive MS patients which were available for this study. The underlying mechanistic pathophysiological processes are currently unclear. It is, however, tempting to speculate that this ratio identifies an AE patient population with predominant compartmentalized neuroaxonal injury (autoantibody‐mediated or cytotoxic) behind a relatively intact blood–brain barrier. In other disease conditions, the use of a ratio instead of single values has also shown interesting results. Specifically, a higher sGFAP/sNfL ratio was reported in active neuromyelitis optica spectrum disorder (NMOSD) patients due to predominantly astrocytic injury compared to MS patients and controls [23]. Similarly, we found a clearer separation of active AE patients from inactive patients when applying the sNfL/sGFAP ratio—compared to the application of mere sNfL or sGFAP values alone. The effect is unique for the serum‐derived ratios because we could not observe it in CSF‐derived ratios, which could be explained by different clearing mechanisms of cGFAP and cNfL. In accordance with our findings, in a large Alzheimer's disease patient cohort, sGFAP also correlated better than cGFAP with disease biomarkers [24].

Limitations of our study are the retrospective design, limited patient number, and heterogeneity of AE patients, not allowing subanalysis for specific antibodies. Further, NfL and GFAP levels are influenced by comorbidities. While age was taken into account in our statistical analysis, *z*‐values are currently only available for serum NfL but not for serum GFAP and CSF values. The future use of comorbidity‐corrected NfL and GFAP values might increase the sensitivity of data analysis.

Beyond the potential for diagnosing AE, the sNfL/sGFAP ratio could prove beneficial in clinical routine as a detection tool identifying patients with an active disease state, especially if MRI scans are negative and CSF results are normal. Interestingly, while sNfL correlated with mRS and CASE, sGFAP only correlated with CASE, a clinical score tailored to the diverse spectrum of AE‐associated neuronal dysfunction. This underscores the utility of GFAP as an additional biomarker in AE. Our results suggest that using the ratio of NfL/GFAP as biomarkers in AE could improve diagnosis and disease activity monitoring in AE.

## Author Contributions

**Johannes Piepgras:** conceptualization, investigation, formal analysis, writing – original draft, visualization, funding acquisition, project administration. **Marlene L. Piepgras:** conceptualization, formal analysis, writing – review and editing, validation. **Falk Steffen:** investigation, writing – review and editing. **Laura Ehrhardt:** investigation, writing – review and editing. **Martin A. Schaller‐Paule:** investigation, writing – review and editing. **Yavor Yalachkov:** investigation, writing – review and editing. **Frauke Zipp:** funding acquisition, writing – review and editing, supervision. **Stefan Bittner:** supervision, project administration, writing – review and editing, validation, funding acquisition.

## Conflicts of Interest

The authors declare no conflicts of interest.

## Data Availability

Raw data used for preparation of figures and tables will be shared in anonymized format on request by a qualified investigator to the corresponding author for purposes of replicating procedures and results.
